# Improving mental health literacy among young people aged 11–15 years in Java, Indonesia: co-development and feasibility testing of a culturally-appropriate, user-centred resource (IMPeTUs) – a study protocol

**DOI:** 10.1186/s12913-019-4328-2

**Published:** 2019-07-12

**Authors:** Helen Brooks, Irmansyah Irmansyah, Karina Lovell, Ira Savitri, Bagus Utomo, Benny Prawira, Livia Iskandar, Laoise Renwick, Rebecca Pedley, Agustin Kusumayati, Penny Bee

**Affiliations:** 1 Department of Health Services Research, Institute of Population Health Sciences, University of Liverpool, Liverpool, UK; 2 National Institute of Health Research and Development, Jakarta, Indonesia; 3 Marzoeki Mahdi Hospital, Bogor, Indonesia; 4 Division of Nursing, Midwifery and Social Work, School of Health Sciences, Faculty of Biology, Medicine and Health, University of Manchester, Manchester Academic Health Science Centre, Manchester, UK; 5 Greater Manchester Mental Health NHS Foundation Trust, Manchester, UK; 6 Marzoeki Mahdi Hospital, Bogor, Indonesia; 7 KPSI, Jakarta, Indonesia; 8 Atma Jaya Catholic University of Indonesia, Jakarta, Indonesia; 9 Into the Light, Jakarta, Indonesia; 10 Indonesian Agency for Witness and Victims Protection, Jakarta, Indonesia; 11 Pulih@thePeak- Women, Youth and Family Empowerment Center, Jakarta, Indonesia; 12 School of Public Health, Universitas Indonesia, Depok, Indonesia

**Keywords:** Mental health literacy, Indonesia, Mental health, Patient and public involvement, Study protocol

## Abstract

**Background:**

Depression and anxiety are two of the leading causes of disease burden in low-to-middle income coutnries. The World Health Organisation has engaged in a programme of scaling-up mental health services, but significant challenges remain. Improving mental health literacy in children and young people, a core part of recent, global health strategies has the potential to address some of these challenges. The study aims to co-develop and feasibility test, a culturally-appropriate toolkit to promote depression and anxiety focused mental health literacy and self-management skills in Indonesia, for children aged 11–15 years.

**Methods:**

A mixed methods study comprising four phases. Through a systematic review of existing evidence, phase 1 will review approaches to improve mental health literacy and self-management in South East Asia and critically review current evidence regarding intervention effect. Phase 2 will explore stakeholders’ views on depression, anxiety and mental health more broadly and identify priorities for the intervention through the use of semi-structured interviews and/or focus groups with policy makers, clinicians, teachers, adolescent service users, carers and young people aged 11–15. Phase 3 will comprise iterative workshops with local stakeholders to present our findings and co-produce a testable, culturally appropriate toolkit to promote mental health literacy and depression/anxiety focused self-management in 11–15 year olds in Java, Indonesia. Phase 4 comprises feasibility evaluation of our developed intervention via nine in-depth case studies (Jakarta, Bogor and Magelang). We will examine the impact, acceptability and feasibility of our prototype intervention and produce evidence-based guidelines for wider implementation.

**Discussion:**

Tools to support mental health literacy and self-management are a low cost way in which mental health services in LMICs can attempt to address the burden of anxiety and depression amongst children and young people. However, this is an underexplored area in Indonesia. Working closely with local stakeholders, this study will design and undertake feasibility evaluation of co-produced mental health literacy and anxiety and depression focussed interactive self-management tools.

This abstract has also been published on the funders website (UK Research and Innovation. Improving Mental Health Literacy Among Young People aged 12-15 years in Indonesia 2019).

**Electronic supplementary material:**

The online version of this article (10.1186/s12913-019-4328-2) contains supplementary material, which is available to authorized users.

## Background

Mental health disorders account for 13% of the global burden of disease, and affect 10–20% of children and young people (CYP) worldwide. Depression and anxiety are two of the leading causes of mental health disability, affecting 6% of adolescents globally each year [1, 2]. Research shows that the risk of depression rises sharply after puberty [3], and that 50 to 70% of depressed adolescents have a recurrent episode within 5 years [3]. Depression in adolescence is associated with more severe and persistent depression in adulthood, poorer physical health and functioning across the lifespan, and an increased risk of suicide [4]. Indonesia meets World Bank criteria for a lower middle-income country (LMIC) and studies estimate that nearly 50% of high school students in Indonesia experience depressive symptoms [5]. National student health surveys suggest that, among Indonesian teenagers, suicidal ideation has a 12-month prevalence of 6.8% [6]. Recent evidence from Indonesia Family Life Survey (IFLS-5) suggests the highest prevalence of depressive symptoms (32.0%) amongst adolescent females [7].

Goal 3 of the United Nations Sustainable Development Goals calls for reducing premature mortality by one third by 2030 through the prevention and treatment of non-communicable diseases and the promotion of mental health and wellbeing [8]. Treatment gaps exceed 75% in most LMICs and this has led to urgent calls to scale up service provision. The WHO’s Mental Health Gap (mhGAP) Action Programme [9], specifies depression as a priority condition, and advocates task-shifting to increase service capacity and integrate mental health services into primary and public healthcare. Evidence has shown that with brief training, non-specialist workers, affected individuals and their families can detect and support people with mental health difficulties [10].

The de-centralisation of mental healthcare has emerged as a promising strategy in LMICs, but significant challenges remain. Traditional beliefs that malicious spirit possession or weak character causes mental illness still persist in South-East Asia, and discrimination towards people with mental health difficulties delays up to 80% of people from receiving or providing effective care [11]. Systematic reviews [12] and disease prevention studies [13] suggest that addressing mental health literacy may be an efficacious strategy for reducing local and global health disparities.

Mental health literacy is defined as ‘knowledge and beliefs about mental disorders which aid their recognition, management or prevention.’ [14] It includes i) the ability to recognise disorders and facilitate help-seeking; ii) awareness of the types of professional help and treatments available, iii) knowledge of effective self-help strategies; iv) knowledge and skills to give ‘first-aid’ and support to others; and v) knowledge of how to promote mental wellbeing and prevent mental health disorders [14].

Inadequate mental health literacy in adolescents, often identified in LMICs including Indonesia [15] significantly increases the risk of developing moderate-severe depression [13]. Encouragingly, adolescents demonstrate a strong preference for peer and family support over professional help-seeking strategies, suggesting that universal mental health literacy programs may have benefit for both primary and secondary disease prevention [16]. School-based psycho-educational interventions have been effective in reducing stigma, promoting young peoples’ mental health knowledge, and increasing mental health literacy in higher and lower income countries [17].

Conceptual frameworks identify health literacy as a critical mediator of health and functional outcomes [18]. Systematic reviews and effectiveness studies demonstrate that mental health promotion interventions, when implemented effectively, can have lasting, positive effects on health [19, 20]. Longitudinal, population-based cohorts (*N* = 7857) have demonstrated a relationship between lower mental health literacy and higher mortality rates in older adults [21] and identified mental health literacy as a significant predictor of psychological and pharmacological treatment engagement [22]. Reductions in morbidity and mortality will be mediated by individual, social and system-level variables, especially those that increase health behaviour and/or health service engagement.

Adolescents represent an important social and demographic group in the WHO South-East Asia Region, accounting for almost one fifth (362.2 million individuals) of the population [6]. Mental health problems in young people are therefore not only a major public health challenge in this region, but also a significant developmental issue and thus a promising point for intervention. Rates of under-diagnosis and under-treatment of depression and anxiety are higher in adolescents than in adult populations, resulting in poorer clinical and social outcomes for those who do not receive appropriate intervention [23]. This may be particularly so in LIMCs, where limited resources and cultural norms can greatly affect how depression and anxiety are expressed and perceived.

In Indonesia, mental health is a national priority but community-based mental health programmes remain in their infancy. This early stage of development presents a unique opportunity to co-develop mental health literacy resources for young people, ensuring that they support emerging health systems and the needs and preferences of their end-users. This project will develop a simple, low cost approach to improving mental health literacy in young people aged 11–15 years by embedding an interactive group resource into school and community health settings. The study arises directly from consultation with local people including mental health service users, carers, and professionals, who identified a lack of culturally appropriate resources to promote the mental health of children and adolescents in Indonesia. Our primary output (literacy toolkit) aligns closely with WHO recommendations to strengthen mental health education in schools. Our secondary outputs (implementation guidance) will assist local adoption, and inform evidence-based, context- relevant policy actions for adolescent mental health promotion in Indonesia and the South-East Asia Region.

## Methods/design

Using the MRC framework for complex interventions, our mixed methods study will comprise four separate but related phases. Primary data collection will be undertaken by Indonesian co-applicants, researchers and patient and public involvement (PPI) representatives and will take place across three study sites in Indonesia; Jakarta, Bogor and Magalang. These sites were selected due to their differing levels of culture, urbanisation and health service development. These three sites also have child and adolescence mental health clinics, which are a valuable resource for conducting research activities and implementing the toolkit. Analysis will be undertaken collaboratively within the wider research team. The manuscript has been prepared using the Standard Protocol Items: Recommendations for Interventional Trials guidelines (SPIRIT). Data management procedures are available from the authors on request.

### Primary aim

The study aims to co-develop and feasibility test, a culturally-appropriate toolkit to promote mental health literacy and depression/anxiety focused self-management skills in young people, aged 11–15 years, in Java, Indonesia.

### Research objectives

1. Systematically review the existing evidence to:
  - Provide a descriptive overview of interventions used to address mental health literacy and/or depression/anxiety self-management in children and young people (CYP) in South East Asia.
  - Examine the effect of these interventions on mental health literacy levels and depression/anxiety self-management skills, and explore where possible, potential associations between intervention delivery and effect.
  - Examine possible factors influencing the uptake and acceptability of these interventions, including barriers/enablers to their implementation.
2. Explore current understanding and perceptions of depression, anxiety and mental health generally amongst CYP in Java, Indonesia and ascertain stakeholder priorities for intervention.
3. Synthesise our learning from phase 1 and 2 to co-produce, with CYP, parents and professionals, an evidence-based, culturally-appropriate toolkit to promote mental health literacy and depression/anxiety focused mental self-management skills in young people aged 11–15 years in Indonesia.
4. Train intervention facilitators and deliver our intervention in nine study sites, purposively selected to represent different health systems and implementation contexts.
5. Evaluate the feasibility and acceptability of our intervention from the perspective of CYP, their parents and families, and health and education professionals.
6. Refine our intervention, and develop best-practice guidance to optimise intervention delivery and engagement.
7. Formulate evidence-based recommendations for future research and practice, and collaboratively develop a subsequent grant proposal.

#### Phase 1: systematic review (months 1–9)

##### Aim

We will undertake a rapid evidence synthesis to i) identify the range of approaches that have been used to address mental health literacy and/or depression/anxiety self-management in children and adolescents in South East Asia, ii) determine their effect and iii) identify potential factors and delivery characteristics influencing their effect, acceptability and implementation.

Details of the review can be found on PROSPERO [https://www.crd.york.ac.uk/prospero/display_record.php?RecordID=108883 – PROSPERO 2018 CRD42018108883].

##### Method

Our mixed-methods review will include published quantitative and qualitative research studies, and unpublished grey literature (e.g. relevant work undertaken by NGOs).

##### Search strategy and data sources

PsycINFO, MEDLINE, Embase, Cochrane Central Register of Controlled Trials (CENTRAL), Scopus, Cumulative Index to Nursing and Allied Health Literature Plus (CINAHL Plus), Social Sciences full texts, ASSIA, ERIC, SCI and SSCI will be systematically searched for relevant publications. Reference checking, targeted author searches and forward-citation tracking will also be conducted. Pluye et al’s scoring system, suitable for qualitative, quantitative and mixed methods research, will be used to assess the quality of included studies [24].

##### Eligibility criteria

We will include all publications published in English and/or local languages e.g. Bahasa, undertaking direct translation where necessary. We will include psycho-educational interventions delivered in any health/community setting, with a primary focus on mental health literacy or depression/anxiety self- management. Eligible populations will include children and young people under 18 years (or where the mean age is < 18) with or without pre-existing mental health conditions, who live in south-east Asia. Primary outcomes will comprise subjective or objective measures of mental health literacy, self-management skills or knowledge. Secondary outcomes will include health beliefs and attitudes, self-esteem, mental health symptoms and quality-of-life.

##### Eligibility assessment

Results from database searching will be uploaded to Endnote before exporting to data management software Covidence (www.covidence.org). Duplicates will be removed before the review process starts. The first stage of screening will involve screening by two independent reviewers at the level of title and abstract. Inclusion/exclusion conflicts will be resolved by a third reviewer. Next, full texts will be reviewed by two reviewers, with conflicts resolved by a third reviewer.

##### Grey literature

A detailed grey literature search protocol (available from the author) will be developed based on local expertise of study partners in the South-East Asian area. Searches will be undertaken across grey literature databases (e.g Open grey, WHO Iris database) and internet search engines (Google).

##### Data extraction

Data extraction templates will be populated with details of the study context (e.g. country), participant sample, intervention, design/content and intervention outcomes. Two reviewers will independently extract the first five studies. If extractions sufficiently match, the remaining studies will be extracted by one reviewer. Any discrepancies will be resolved by consensus.

##### Data synthesis

If data allows, we will conduct meta-analyses using random-effects modelling to provide measures of pooled effects and a meta-regression of potential effect moderators to examine associations between intervention components and outcomes. Where data is insufficient or unsuitable for meta-analysis, we will conduct a narrative synthesis.

Our findings will be distilled and tabulated into a thematic framework cross-referencing intervention content and delivery characteristics against intervention reach, acceptability and outcome. Review findings will be integrated with primary research data collected in Phase 2 to inform the development an evidence based, mental health toolkit to improve mental health literacy and depression/anxiety self-management in children and young people in Indonesia in phase 3. We will work with our project advisory panel to review this evidence and to select additional patient-prioritised outcomes for our evaluation.

#### Phase 2: stakeholder interviews/focus groups (months 4–10)

##### Aim

Phase 2 aims to explore through primary research understanding and perceptions of mental health and depression and anxiety in particular amongst children and young people in Java, Indonesia. It will also explore key stakeholder (CYP, teachers, health professionals and national level stakeholders relevant to policy and practice development) priorities for intervention.

##### Methods

Sampling and recruitment.

Participants must belong to one of the following groups:

- Child or young person aged 11–15 with or without depression and anxiety
- Parent of a child age 11–15 with depression and/or anxiety
- Professionals involved in the care of young people age 11–15 such as clinicians, teachers
- Key informants whose role at a national or local level is likely to influence the education/care of children aged 11–15 e.g. government ministers, policy makers, service directors, senior management and community leaders.

Participants must be able to give informed consent to Indonesian researchers. In the case of children/young people, assent of the young person and consent of their parent/guardian will be required (see Additional file 1 for an example participant information sheet and consent form).

We will aim to purposively sample 15–20 children and young people (non-service users) based on age, gender, and geographical area. An additional 15–20 children and young people with depression or anxiety (current mental health service-users) and 15–20 parents/carers of children and young people with depression and/or anxiety will also be purposively sampled (on age, gender, geographical location and time since diagnosis), recruited through primary care services and CAMHS.

We will additionally aim to recruit and interview mental health professionals, community mental health workers and teachers for their views on intervention design (*n* = 10–15 in each professional group). We will supplement these data with key informant interviews (*n* = 8–10), identified via study team contacts and purposive sampling, to illuminate and explore broader influences on intervention implementation. Key informant interviews will include, at national level, government ministers, policy makers, and leaders of third sector organisations, and at a local level, service directors, senior management and community leaders. Interview schedules will be developed drawing on findings from Phase 1, Jorm et al’s definition of mental health literacy [14] and consultation with our PPI advisory group.

The study will be promoted through posters and existing community networks and social media channels identified by local collaborators. Relevant health and education professionals will distribute details on the study including an invitation letter and patient information sheet.

##### Data collection

We will use qualitative, semi-structured interviews or focus groups to collect data. Interviews with children with incorporate a photo elicitation method, to encourage discussion of a potentially sensitive topic. Photo elicitation methods (e.g. asking participants to take photographs visually portraying ‘mental health’ and then narrating the meaning of photos in subsequent qualitative interviews/focus groups) have been successfully used within mental health research [25] and can facilitate researcher-participant relationships by increasing participant empowerment and providing participants with control over the research process [26]. Participants who do not have access to a smartphone will be provided with one to allow them to take photographs prior to interview. Photographs will form a unit of analysis to support emerging themes and as a research and dissemination tool in Phase 3.

Data collection will explore beliefs, attitudes and experiences of mental health, including adolescent depression and anxiety where relevant. The schedule is organised around the various components of mental health literacy [14]. Participants’ priorities and preferences for intervention design and format will also be explored.

##### Data analysis

All interviews/focus groups will be conducted by researchers from Indonesia, who will be provided with training and supervision from the study team. Transcripts will be translated into English and independently validated by a bilingual individual. A proportion (5%) will be back translated to ensure correct interpretation [27]. Analysis: A six-stage thematic analysis [28] will be conducted, supported by NVivo software. Transcripts will be independently coded by Indonesia researchers, with data interpretations discussed and verified among the wider study team.

#### Phase 3: co-production workshops and prototype resource development (months 11–19)

##### Aim

Phase 3 will co-produce, with key stakeholders, an evidence-based, culturally appropriate toolkit to promote depression and anxiety focussed mental health literacy amongst children and adolescents aged 11–15 years in Java, Indonesia.

##### Methods

Using data from phase 1 and 2, intervention resources will be designed, in Indonesia, in collaboration with a group of children and adolescents (*n* = 8–10) and adult carers, designers and health and education professionals (*n* = 8–10). We will use a framework for experience-based co-design developed by Kings College London which involves initial design workshops, smaller sustained group work and final review events [29].

PPI consultation to date has suggested an interactive, group intervention. Group-delivery is a low-cost delivery model which can derive additional benefits through peer-to-peer support. Systematic review and meta-analysis [30, 31] suggests that engaging students in activities such as games, simulations and group work is more effective than relying solely on didactic methods. We will align the structure of our intervention with data from phases 1–2 and current evidence [32–34] supporting the effectiveness of short-term programmes (max 8–9 h over 3–8 sessions) for mental health education and mental health first-aid. Frequency, duration and content of the intervention will be recorded by facilitators and reviewed in Phase 4 facilitator and service-user interviews to explore whether treatment was delivered as intended.

We will conduct up to 6 half-day stakeholder consultation events (3 per group), comprising of presentations, discussion of Phase 1 and 2 findings and mixed small and large group activities, using age-appropriate creative methods, to identify an appropriate implementation model and develop culturally-relevant resources. Components will be derived from Phase 1 learning, with group consensus informed by RAND Appropriateness Methodologies [35]. A recent systematic review identifies local consensus processes as an effective method of promoting the uptake of evidence-based interventions into practice [36].

##### Analysis

Our workshop outputs from will take the form of a logic model, outlining the inputs, preferred activities, outputs and impacts of the intervention. Designers will work with the outputs from the consultation, and child and adolescent generated visual data, to design prototype resources. We will document our intervention according to the template for intervention description and replication (TIDieR) checklist [37], and supplement our resources with an evidence-based framework (e.g. implementation guidance, intervention facilitator training and a half-day train-the- trainers workshop) to support their longer-term sustainability and facilitation.

#### Phase 4: prototype testing and evaluation case studies (months 20–30)

The MRC recommends feasibility testing to ensure new interventions can be implemented. Utilising outputs from Phases 1–3, Phase 4 of our study will test the content, format and implementation of our prototype intervention. We will use a comparative case study approach which is recommended when it is not feasible and/or too premature to conduct studies of an experimental design. The approach produces testable knowledge about causal pathways (e.g. how and why our co-developed intervention works or fails in different contexts) for subsequent exploration in a future feasibility trial.

##### Aim

Phase 4 will evaluate the acceptability and feasibility of a co-developed depression and anxiety self-management intervention for 11–15 year olds in Java, Indonesia.

##### Methods

Utilising data collected during phases 1–3 of the study, our intervention will be tested in nine sites in Jakarta, Bogor and Magalang (one CAMHs service, one school and one community health team in each setting). We will use a collective case study design [38] in three geographical areas. Our sites have been purposively sampled to include diversity in terms of geographical area, urban/rural/sub-urban populations, levels of mental health service provision, and cultural aspects. In line with recent guidance [39], we will appoint local opinion leaders at each implementation site and draw on this social influence to engage practitioners and educational facilitators in our intervention. We will use the MRC process evaluation model [40] to explore the delivery and reach of our intervention, and understand barriers/enablers to its rollout.

In-depth mixed-methods implementation case studies will be undertaken at each site including semi-structured qualitative interviews with key stakeholders (*n* = 30 users, carers and professionals; total interview number across all sites = 270). We will collect quantitative, site-specific data on intervention uptake, reach and impact.

Quantitative children and young people’s outcome measures will be completed at baseline, post- intervention and 6-month follow-up for feasibility analysis. The primary outcome for a future definitive trial of our intervention is anticipated to be an adapted version of the mental health literacy scale [MHLS, [41]]. Additional measures will include the Reynolds Adolescent Depression Scale [RADS, [42]] which has been used previously with Indonesian populations, a culturally appropriate service use questionnaire, the Family adaptability and cohesion scale (FACESII), which has been validated for use within Indonesia children and adolescents [42] and the validated Indonesian version of the SF-36 quality of life questionnaire [43]. These will be supplemented by additional measures identified via our phase one review and prioritised by our PPI advisory group. We will assess the feasibility of these different outcome measures as part of our case study evaluation.

##### Analysis

As this is a feasibility evaluation, our quantitative analysis will be mostly descriptive. We will monitor the number of children and young people, parental and facilitator consents to research participation and cumulative and monthly recruitment and retention rates. We will examine intervention uptake and delivery rates and compare the proportion of children and young people who engage in the intervention at different sites and via different implementation routes.

Descriptive statistics will assess the completeness and variability of outcome measures at each data collection point, including potential floor and ceiling effects. To inform subsequent research, we will undertake exploratory comparisons of intervention outcomes on an intention-to-treat basis, recognising that these analyses may be underpowered. We will synthesise our qualitative findings with our quantitative data to hypothesise if and how inequalities in intervention reach and outcome arise.

### Patient and public involvement

We will establish a study advisory panel independent of the project team with support from project partners. This panel will be based in Indonesia and will comprise 10–12 key stakeholders including young people, parents, health and education professionals and third sector representatives. Based on our prior experience of working with children and young people, 2 advisory sub- panels will be established: one for children and young people (*n* = 4), and one for adults (*n* = 6–8). Sub-panels will meet be-annually throughout the project. To facilitate communication between the two sub-panels, a volunteer representative from the children and young people’s panel will join adult panel meetings.

We have designed a training/development strategy for all PPI representatives on our programme. This course is cited as good practice by the Mental Health Research Network and included in the NICE shared learning database [44]. Training will enable service users, carers and other PPI partners to i) be involved in all stages of the project, ii) co-produce our intervention and implementation strategies, iii) co-produce and co-deliver dissemination materials and iv) co- develop future grants.

### Project partners

KPSI is an NGO based in Jakarta, Indonesia, which provides information and support to people living with mental illness and their families. Into the Light, Indonesia is a youth community charity whose work focuses on evidence and rights based suicide prevention and mental health promotion for children and young people and other high-risk groups in Indonesia. The Pulih Foundation develops and delivers community based recovery services for individuals and families. All three organisations will provide advice and feedback over the course of the project to ensure it is conducted in a culturally sensitive manner, that the study addresses the needs of service users and carers and that the study reflects the experiences of local communities.

Partner organisations including the School of Public Health in the University of Indonesia have strong links with local services, service users and communities which will be used to support data collection and recruitment for the study. They will provide expert advice and feedback throughout the project and ensure the study reflects the current evidence base and will help maximise public and community impact and cross-cultural comparative work and psycho- educational dissemination in the UK.

### Dissemination

A core deliverable from our programme will be an interactive, evidence-based toolkit to enhance mental health literacy in young people in Indonesia. In addition to the publications in peer-reviewed journals, we will work closely with voluntary agencies to disseminate our learning and include evidence-based, age-appropriate psycho-education into existing and future programmes. At the end of the project, we will host a one-day mixed-stakeholder dissemination conference in Java to engage a national audience in our research, provide information on our intervention, and encourage wider rollout of our programme deliverables and will host two exhibitions (UK and Indonesia) of photos included in stage 2 of the study.

New knowledge generated by the study will be synthesised and used to underpin the development of a new mental health literacy toolkit (provisionally including a family board game) and accompanying implementation guidance. To maximise impact and reach, we will make all our training and intervention resources freely available to Indonesian and UK health services and third-sector equivalents. To the best of our knowledge, these deliverables are novel and represent new resources of direct relevance to Indonesian health services and equivalents.

## Discussion

Our mixed method study developed collaboratively with Indonesian academics, health professionals and PPI representatives will deliver theoretical, empirical and experiential knowledge to inform and optimise health policy development. We will benchmark young people’s mental health literacy in Java, and advance current understandings of the implementation and cultural acceptability of mental health literacy interventions in Indonesia. We will draw on effective knowledge mobilisation to ensure this information is available to policy makers to underpin new public health strategies and person-centred health policy and care.

By the end of our 30-month study, we will have delivered a testable, culturally-acceptable toolkit to enhance depression and anxiety focussed recognition and management among children and young people in Indonesia. We will have qualitatively explored barriers and enablers to toolkit implementation and engagement, and developed evidence- informed best-practice guidelines to optimise its impact and reach. We will produce a minimum of two subsequent grant applications which will build on this work, including a protocol for a rigorous evaluation of the clinical and cost-effectiveness of our developed intervention. Via our proposed project collaborations, we will have enhanced civic (PPI) engagement in research and built a strong Indonesian research group, with the knowledge and experience required to lead this work.

Our research necessitates the involvement of a range of stakeholders, including children and young people, and focuses on a potentially sensitive research topic. It is possible that recruitment may be influenced by local cultures including negative social representations, prejudice and discrimination toward people with mental illness. To overcome these recruitment barriers, we will work with our advisory panels to develop a bespoke engagement strategy to target children and young people, service users/carers, health and education professionals, policy makers and community and third-sector networks. Direct liaison with local communities has already been initiated through awareness talks in partnership with a local school and national voluntary organisations. Information about the study in the form of social and mainstream media coverage, community posters, and information leaflets in local dialects and languages will be available. All data will be collected by Indonesian researchers in Bahasa Indonesia to ensure the research is sensitive to local cultures and customs. We will draw on existing evidence to facilitate intervention uptake and delivery in practice [45] and, via our collaborators in Indonesia, build meaningful and enduring research partnerships with local health services, schools and community groups.

### Strengths and limitations

The study gains is strengthened by the existing partnership between UK and Indonesian collaborators, the network of project partners aligned with the study, centrality of PPI to the design and undertaking of the study, and the in-depth nature of the methods. The feasibility evaluation will only recruit participants from three geographical locations within Java (Jakarta, Bogor and Magalang). Results are therefore unlikely to be generalizable to participants in other areas of Indonesia.

The systematic review is focussed on South East Asia which as a geographical area includes countries such as Singapore which is not considered to be a LMIC and may differ in important contextual ways to other South East Asian countries.

## Additional file

Additional file 1: Example information sheet and consent form. (DOCX 122 kb)

## Data Availability

Not applicable for a protocol paper. All investigators will have access to final datasets. Anonymous data can be deposited in relevant repositories and will be available on request from the authors.

## Abbreviations

CAMHS: Child and adolescent mental health service
CYP: Children and young people
FACES: Family adaptability and cohesion scale
KPSI: Komunitas Peduli Skizofrenia Indonesia
LMIC: Low-Middle Income countries
mhGAP: Mental Health Gap
MHLS: Mental health literacy scale
NGO: Nongovernmental organisations
NICE: National Institute of Clinical Excellence
PPI: Patient and public engagement
RADS: Reynolds Adolescent Depression Scale
SEA: South East Asia
SPIRIT: Standard Protocol Items: Recommendations for Interventional Trials guidelines
UK: United Kingdom
WHO: World Health Organisation
