# Supplementary material for: Improving mental health literacy among young people aged 11–15 years in Java, Indonesia: co-development and feasibility testing of a culturally-appropriate, user-centred resource (IMPeTUs) – a study protocol
Source: BMC Health Serv Res. 2019 Jul 12;19:484. doi: 10.1186/s12913-019-4328-2 (PMC6626417; doi:10.1186/s12913-019-4328-2)
Supplement: Supplementary file 1 — Example information sheet and consent form. (DOCX 122 kb) [file 12913_2019_4328_MOESM1_ESM.docx]

Additional File 1: Example Information Sheet and Consent Form

**Improving Mental Health Literacy Among Young People aged 11-15 years in Indonesia: IMPeTUs**

**Participant Information Sheet (PIS) – parent interviews/focus groups**

This PIS should be read in conjunction with the University of Manchester privacy notice (see attached).

You are being invited to take part in a research study investigating ways to help young people living in Indonesia to look after their mental health. Before you decide whether to take part, it is important for you to understand why the research is being conducted and what it will involve. Please take time to read the following information carefully and discuss it with others if you wish. Please ask if there is anything that is not clear or if you would like more information. Take time to decide whether or not you wish to take part. Thank you for taking the time to read this.

**Who will conduct the research?**

The study has been developed by a team of researchers and clinicians from the University of Manchester (UK), the University of Liverpool (UK) and the University of Indonesia.

**What is the purpose of the research?**

Many young people suffer from mental health problems, such as depression and anxiety. We want to develop a support resource (or ‘toolkit’) that will give young people the skills and knowledge they need to look after their mental health. Before we do this, we need to speak to young people and those who support them (such as parents and teachers) to understand what the toolkit needs to include, how it should look and how it should be delivered. This will help us to make sure that the toolkit will bring real benefit to young people living in Indonesia.

**Why have I been chosen?**

You have been invited because you are the parent/carer of a young person aged 11-15 years who has experience of depression or anxiety. Alternatively, you might have seen a study flyer and are interested in taking part.

In total, we want to speak to about 20 parents/carers, 40 young people and about 40 professionals, such as teachers and health professionals.

**What would I be asked to do if I took part?**

If you contact us to express interest in the study, a researcher will arrange a convenient date and time to meet you. This meeting will usually take place at your home or another community venue.

Taking part involves taking part in a single interview/focus group with a researcher. We will tell you when you first talk to the interviewer whether we are asking you to take part in a group discussion (a ‘focus group’) or a one-to-one interview. If you take part in a focus group, 7-9 other parents will take part. We know that group discussion can be helpful as hearing other people’s ideas can get people to think more deeply about the topic. If you would prefer to meet with the researcher one-to-one, just let the researcher know and they will be able to arrange this.

Before the interview/focus group, the researcher will tell you about the study and give you the chance to ask any questions. If you want to take part, you will be asked to sign a consent form. You are free to say no without giving a reason.

If you consent to taking part, we will ask you to fill in a short questionnaire to record basic information about you and your child.

The researcher will then ask some questions about your understanding of mental health and how you think we should design our toolkit. We will record the sound so that the researcher has an accurate and detailed record of your interview/focus group.

**What will happen to my personal information?**

In order to undertake the research project we will need to collect the following personal information/data about you:

- Your name and address (for the purposes of arranging the /focus group and letting you know about study results).
- An audio-recording (voice only) of the interview/focus group. We will write up this recording word for word so that the research team can analyse the information.

Only the research team will have access to this information.

We are collecting and storing this personal information in accordance with the General Data Protection Regulation (GDPR) and Data Protection Act 2018 which legislate to protect your personal information. The legal basis upon which we are using your personal information is “public interest task” and “for research purposes” if sensitive information is collected. For more information about the way we process your personal information and comply with data protection law please see our [Privacy Notice for Research Participants](http://documents.manchester.ac.uk/display.aspx?DocID=37095).

The University of Manchester, as Data Controller for this project, takes responsibility for the protection of the personal information that this study is collecting about you. In order to comply with the legal obligations to protect your personal data the University has safeguards in place such as policies and procedures. All researchers are appropriately trained and your data will be looked after in the following way:

The study team will have access to your personal identifiable information, that is data which could identify you, but they will anonymise it as soon as practical. However your consent form, contact details, etc will be retained securely (separated from your data) until the end of the project, then securely destroyed.

Any data transferred by our Indonesian research team to the University of Manchester research team will be transferred using a secure electronic service called Dropbox for Business. Any personal digital data (data which identifies you, such as your consent form) transferred will be encrypted and password protected to make sure others cannot access it. We will make your fully anonymised data publically accessible so that others may use the research data.

You have a number of rights under data protection law regarding your personal information. For example you can request a copy of the information we hold about you, including audio recordings. This is known as a Subject Access Request. If you would like to know more about your different rights, please consult our [privacy notice for research](http://documents.manchester.ac.uk/display.aspx?DocID=37095) and if you wish to contact us about your data protection rights, please email [dataprotection@manchester.ac.uk](mailto:dataprotection@manchester.ac.uk) or write to The Information Governance Office, Christie Building, University of Manchester, Oxford Road, M13 9PL. at the University and we will guide you through the process of exercising your rights.

You also have a right to complain to the [Information Commissioner’s Office](https://ico.org.uk/concerns), Tel +44 303 123 1113

**Will my participation in the study be confidential?**

Your participation in the study will be kept confidential, with access to your personal information restricted to the study team. The only exception to this would be if you share something with us which reveals that someone is at risk of harm. In this case, we may be required to act on this information but we would not do this without involving you in the process. Individuals from the University of Manchester, University of Liverpool and University of Indonesia where the research is taking place and regulatory authorities may need to review the study information for auditing and monitoring purposes or in the event of an incident.

Audio-recordings will be made using an encrypted, password protected device that people outside the team will not be able to access. Once the recording is made, it will be transferred to a password protected, encrypted computer that only people in the team can access. As soon as these files are received, they will be labelled with an identifier (a ‘key’). A list linking your name to the identifier will be kept securely, separate from your transcript. Only the immediate team will have access to the list that links you to the data.

Members of the team in Indonesia will transcribe (write out word for word) your interview/focus group. The team will make sure the transcriptions are fully anonymised (personal information will be removed). Once the interview/focus group is transcribed, the audio-recording will be destroyed.

Anonymised transcripts will be transferred securely to researchers at the University of Manchester and University of Liverpool through a service called Dropbox for Business. Consent forms will be sent in the same way but will be encrypted and password protected so that no one outside the study team would be able to access the file.

The UK teams will download your data and keep it confidentially on an encrypted password protected server. Your data will be stored in the same way so that it is always labelled with an ID number stored separately from your name.

We will destroy your personal details at the end of the study. Anonymised data (transcriptions) will be retained for a minimum of 10 years.

**What happens if I do not want to take part or if I change my mind?**

It is up to you to decide whether or not to take part. If you do decide to take part you will be given this information sheet to keep and be asked to sign a consent form. If you decide to take part you are still free to withdraw at any time without giving a reason and without detriment to yourself. If you take part in a one-to-one interview, you will be able to withdraw your data for up to two weeks. After two weeks, it will not be possible to remove your data. If you take part in a focus group, we will unfortunately be unable to remove your data, as it will be mixed together with other people’s data. This does not affect your data protection rights.

Unfortunately, you cannot take part in this study if we do not have your consent to audio-record the interview/focus group. This is because other people within the team (who will not be present during the interview/focus group) need to look at the data. You can stop the recording or leave the focus group at any time if you decide you are not comfortable with the recording.

**Will my data be used for future research?**

When you agree to take part in a research study, information about you may be provided to researchers running other research studies in this organisation. The future research should not be incompatible with this research project and will concern research to improve the mental health of young people in Indonesia. These organisations may be universities, NHS organisations or companies involved in specific relevant research in this country or abroad. Where your information relates to your health and care it will only be used by organisations and researchers to conduct research in accordance with the [UK Policy Framework for Health and Social Care Research](https://www.hra.nhs.uk/planning-and-improving-research/policies-standards-legislation/uk-policy-framework-health-social-care-research/).

This information will not identify you and will not be combined with other information in a way that could identify you. The information will only be used for the purpose of health and care research, and cannot be used to contact you regarding any other matter or to affect your care. It will not be used to make decisions about future services available to you.

Please note, with your consent, we will add your name and contact details to a register to receive information about the progress/findings of this research. If you do not want to receive this information, just leave this box on the consent form blank. We will destroy your details at the end of the study.

**Will I be paid for participating in the research?**

We would like to offer you a small payment (Rp50,000) to say thank you for your involvement. We can reimburse costs involved if you have to travel to meet the research team.

**What are the benefits of this research?**

This study may not help you personally, but the findings will help researchers to develop a toolkit to support other young people living in Indonesia in the future.

**What is the duration of the research?**

Taking part in a one-to-one interview will take about 60-90 minutes of your time. If you take part in a focus group, the meeting may last around 90-120 minutes.

**Where will the research be conducted?**

The researcher will arrange to meet you at a location that is convenient for you, such as your home or other place in your local community.

**Will the outcomes of the research be published?**

At the end of the research, the results will be made available in reports and academic papers. A summary of findings will be sent to you. When we write up the results, all personal details will be removed so that no-one will know who you are. We may use direct quotes from the meeting but no real names will be used.

**Who has reviewed the research project?**

This research has been reviewed by the University of Manchester Research Ethics Committee. It has also been approved by the Ministry of Health in Indonesia.

**What if I want to make a complaint?**

*Minor complaints*

If you have a minor complaint then you need to contact the researcher(s) in the first instance:

NAME OF INDONESIAN RESEARCH CONTACT

PROFESSIONAL EMAIL ADDRESS

PROFESSIONAL PHONE NUMBER

ADDRESS

*Formal Complaints*

If you wish to make a formal complaint or if you are not satisfied with the response you have gained from the researchers in the first instance then please contact

The Research Governance and Integrity Manager, Research Office, Christie Building, University of Manchester, Oxford Road, Manchester, M13 9PL, by emailing: [research.complaints@manchester.ac.uk](mailto:research.complaints@manchester.ac.uk)  or by telephoning +44 161 275 2674.

**What Do I Do Now?**

If you have any queries about the study or if you are interested in taking part then please contact the researcher(s):

NAME OF INDONESIAN RESEARCH CONTACT

PROFESSIONAL EMAIL ADDRESS

PROFESSIONAL PHONE NUMBER

ADDRESS

***Services offering help and support***

Recovered @ the peak

Jl. Brawijaya Raya No.15, RT.3 / RW.3, Pulo, Kby. Baru, South Jakarta City, Jakarta Capital City 12160.

Phone (021) 72798747

The community cares about Indonesian schizophrenia

Jl. Raya Jatinegara Timur No.99 Kel, RT.9 / RW.2, Bali Mester, Kec. Jatinegara, East Jakarta City, Special Capital Region of Jakarta 13300.

Phone (021) 8579618

Komnas Perempuan Jl.

Lembang No.4B, RT.1 / RW.4, Menteng, Central Jakarta City, Jakarta Special Capital Region 10310.

Phone (021) 3903963

**This Project Has Been Approved by the University of Manchester’s Research Ethics Committee [2018-4949-7908] and the Ministry of Health (Indonesia) Ref: LB:02.01/2/KE.201/2019**

**Improving Mental Health Literacy Among Young People aged 11-15 years in Indonesia: IMPeTUs**

**Consent Form – phase 2 – parents’ interviews/focus groups**

If you are happy to participate please complete and sign the consent form below

|  | **Activities** | Initials |
| --- | --- | --- |
| 1 | I confirm that I have read the attached information sheet (**Version XX, Date dd/mm/yyyy**) for the above study and have had the opportunity to consider the information and ask questions and had these answered satisfactorily. |  |
| 2 | I understand that my participation in the study is voluntary and that I am free to withdraw at any time without giving a reason and without detriment to myself. If I take part in an individual interview, I understand that I can withdraw my data for up to two weeks after my interview, but after this time my data cannot be withdrawn. If I take part in a focus group, I understand that I will not be able to withdraw my data.  I agree to take part on this basis |  |
| 3 | I agree to my discussions being recorded and transcribed (written out in full) by one of the research team |  |
| 4 | I agree that any data collected, including direct quotes, may be used in anonymous form in publications and presentations. |  |
| 5 | I agree to my data being securely shared from Indonesia to the research team in the United Kingdom. |  |
| 6 | I understand that the anonymised transcript of my interview may be shared with other researchers to support future studies. My transcript will only be shared once all personal information that would identify me has been removed. |  |
| 7 | I understand that data collected during the study, may be looked at by individuals from the University of Manchester or by regulatory authorities, where it is relevant to my taking part in this research. I give permission for these individuals to have access to my data. |  |
| 8 | I agree that the researchers may retain my telephone number in order to provide me with a summary of the findings for this study. |  |
| 9 | I understand that all information obtained during the study will remain confidential unless there are concerns about my safety or the safety of others. In such cases, the researchers will inform the relevant authorities but will always discuss this with me first. |  |
| 10 | I agree to take part in this study |  |

**Data Protection**

**The personal information we collect and use to conduct this research will be processed in accordance with data protection law as explained in the Participant Information Sheet and the** [**Privacy Notice for Research Participants**](http://documents.manchester.ac.uk/display.aspx?DocID=37095)**.**

________________________ ________________________

Name of Participant Signature Date

________________________ ________________________

Name of the person taking consent Signature Date

1 copy for the participant, 1 copy for the research team (original).
